# Weight changes and lifestyle behaviors in women after breast cancer diagnosis: a cross-sectional study

**DOI:** 10.1186/1471-2458-11-309

**Published:** 2011-05-13

**Authors:** Yong Heng Yaw, Zalilah Mohd Shariff, Mirnalini Kandiah, Chan Yoke Mun, Rokiah Mohd Yusof, Zabedah Othman, Nurfaizah Saibul, Yong Heng Weay, Zailina Hashim

**Affiliations:** 1 Department of Nutrition and Dietetics, Faculty of Medicine and Health Sciences, Universiti Putra Malaysia, Serdang 43400 Selangor, Malaysia; 2 Cancer Education & Services Laboratary, Institute of Social Science Studies, Universiti Putra Malaysia, Serdang 43400 Selangor, Malaysia; 3 Department of Radiotherapy and Oncology, Hospital Kuala Lumpur, 50586 Kuala Lumpur, Malaysia; 4 Department of Community Health, Faculty of Medicine and Health Sciences, Universiti Putra Malaysia, Serdang 43400 Selangor, Malaysia

## Abstract

**Background:**

Weight gain rather than weight loss often occurs after breast cancer diagnosis despite breast cancer survivors frequently reported making healthful lifestyle changes. This study describes the prevalence and magnitude of changes in weight before and after breast cancer diagnosis and examines lifestyle behaviors of breast cancer survivors with stable weight, weight gain or weight loss.

**Methods:**

Respondents were 368 women with breast cancer characterized by stages I, II and III. All were recruited from hospitals or breast cancer support groups and had completed conventional treatment. Current weight and height were measured while weight at cancer diagnosis and 1 year before diagnosis were self-reported. Weight change was calculated as the difference between current weight and weight a year preceding breast cancer diagnosis. A 24-hour diet recall and Global Physical Activity Questionnaire assessed dietary intake and physical activity, respectively. Differences in lifestyle behaviors among weight change groups were examined using Analysis of Covariance (ANCOVA).

**Results:**

Mean weight change from a year preceding diagnosis to study entry was 2.73 kg (95% CI: 1.90-3.55). Most women (63.3%) experienced weight gain rather than weight loss (36.7%) with a higher percentage (47.8%) having at least 5% weight gain (47.8%) rather than weight loss (22%), respectively. Compared to other weight change groups, women in >10% weight gain group had the lowest fruit and vegetable servings (1.58 servings/day; 95% CI: 1.36-1.82) and highest servings of dairy products (0.41 servings/day; 95% CI: 0.30-0.52).

**Conclusions:**

Weight gain was evident in this sample of women after breast cancer diagnosis. Information on magnitude of weight change after breast cancer diagnosis and lifestyle behaviors of breast cancer survivors with varying degrees of weight change could facilitate the development and targeting of effective intervention strategies to achieve healthy weight and optimal health for better survival.

## Background

Breast cancer is the second most frequent cancer in the world, with an estimated 1.05 million cases in the year 2000 [1]. Breast cancer is also the most commonly diagnosed cancer among Malaysian women. Although the incidence of breast cancer in Malaysia is much lower compared to European countries, it is increasing steadily as it comprised 30.4% of all female cancers [2]. In 2006, 3,525 new cases of breast cancer were reported to the National Cancer Registry (NCR) of Malaysia, giving an age-standardized incidence rate of 39.3 per 100, 000 women [3]. The 5-year survival of European women diagnosed in the period 1990-1994 was 77% [4]. A higher figure of 90% was reported in the United States [5]. However, the overall 5-year survival from breast cancer reported for Malaysian women was 58% between 1993 and 1997 [6].

Weight change occurs in most women after breast cancer diagnosis and the amount of weight gain or loss could vary among the survivors [7]. Most cancers cause weight loss, changes in appetite and dietary intake, reduced physical activity and deterioration of psychological well being. However, weight gain rather than weight loss has been frequently reported in patients after diagnosis of breast cancer. Halbert et al., (2008) [8] found that 47% of women reported weight gain, 32% weight loss and 21% no weight change. Studies have reported a weight gain ranging from 1.0 to 6.0 kg during the first year after being diagnosed with breast cancer [9-11]. However, greater gains are not uncommon [12].

Lifestyle change is one of the many possible actions women may take when they have been diagnosed with breast cancer in order to improve breast cancer prognosis and reduce the probability of cancer recurrence. Several studies [13-15] have found that breast cancer survivors changed their diets with the most common dietary changes reported were decreased consumption of dietary fat and increased intake of fruits and vegetables. Lower energy and macronutrient intakes post-diagnosis have also been reported in response to breast cancer diagnosis [16]. Besides making healthful dietary changes, breast cancer survivors were also more likely to increase or maintain their moderate to vigorous physical activity post-diagnosis [17-19].

As many developing countries, including Malaysia, have disproportionately high rates of breast cancer morbidity and mortality, a comprehensive assessment of lifestyle behaviors after breast cancer is essential as these behaviors may potentially affect prognosis, cancer recurrence, long-term survival and risk of other chronic diseases such as cardiovascular, hypertension and diabetes [11]. To date, information on lifestyle behaviors after breast cancer diagnosis is mainly derived from studies in western population. As lifestyle changes in response to cancer diagnosis could be influenced by various factors within specific settings, the lifetysle behaviors observed in the developing countries may differ from those reported in western population. Cancer awareness and knowledge, motivation to change, professional and social support, access to healthy food are among factors that may vary by cultures which could potentially influence lifestyle behaviors. This study reports on the prevalence and magnitude of weight changes after breast cancer diagnosis and examines self reported dietary intake and physical activity of breast cancer survivors with various levels of weight change.

## Methods

### Study respondents

This cross-sectional survey was conducted in eight general hospitals and four breast cancer support groups in seven states of Peninsular Malaysia. These states represented the Northern, Central, Southern and East Coast regions of Peninsular Malaysia. Respondents were recruited from oncology or surgical out-patient clinics of general hospitals and breast cancer support groups over a period of 10 months (February to December 2008). Lists of cancer patients attending the out-patient clinics for medical check-up as well as active members of support groups were provided by the hospitals and support groups, respectively. Out of 615 breast cancer survivors screened, 400 women met the selection criteria and were invited to participate in this study. A total of 368 women voluntarily participated in the study, giving a response rate of 92%.

This study employed purposive sampling as the selection of respondents was based on several criteria i.e. women 20-65 years old at the time of diagnosis, diagnosed with cancer of stages 0-III, completed conventional treatments (surgery, chemotherapy, radiotherapy) at least 6 months prior to recruitment, free from cancer recurrence and not currently under any medication (e.g. hormonal pills) that would affect the study outcome. All respondents were interviewed using a pre-tested questionnaire either at the hospitals or premises of breast cancer support groups. The study protocol was approved by the Medical Research Ethics Committee of the Faculty of Medicine and Health Sciences, Universiti Putra Malaysia and the Medical Research Ethics Committee (MREC), Ministry of Health Malaysia. Prior to data collection, informed consent was obtained from all respondents.

### Measurements

#### Dietary intake

A 24-hour recall was used to assess the dietary intakes of the respondents. Portion size, brand names and cooking method were recorded. Standard household measuring cups and spoons were used to assist respondents in portion size estimation. The nutritionist Pro™ software version 2.5 (First Data Bank, USA, 2005) [20] was utilized to analyse the dietary data. Several food databases such as Malaysian Food Composition Tables [21], Singapore Food Facts (1999) [22], ASEAN Food Composition Tables [23] and USDA Food Databases were utilized in addition to those available in nutritionist Pro™. For dishes that were not available in any food database, 2-3 recipes were used to calculate the energy and nutrient content, and the average values were used in dietary analysis. Total energy intake, percentage of energy from macronutrients and the number of servings for grains, meats and legumes, fruits, vegetables and dairy products were calculated.

#### Physical activity

The current physical activity level was measured using the Global Physical Activity Questionnaire (GPAQ) [24]. Women were requested to recall the number of days in the last seven days that they did vigorous intensity activity or/and moderate intensity activity at three major settings (activities at work/home, travel to and from places, recreational activities), as well as the number of hours and minutes per day they did the activities, respectively. The respective total hours for physical activity were calculated and multiplied with the metabolic equivalent (MET) hours per week and the values were then categorized into low, moderate and high.

#### Anthropometric measurements

The weight a year preceding breast cancer diagnosis, was based on self-reported information. All respondents indicated that the reported weights were stable for at least 6 months prior to diagnosis. Current weight and height were measured to the nearest 0.1 kg and 0.1 cm, respectively using TANITA digital weighing scale (TANITA Corporation of America, Inc, United States) and SECA body meter (SECA, British Indicators Ltd, United Kingdom). Body mass index (BMI) was calculated and classified according to the World Health Organization classification [25]. Waist circumference was assessed using a SECA microtoise tape (SECA, British Indicators Ltd, United Kingdom). Waist circumference ≥80 cm for women was considered as at high risk for abdominal obesity [25].

Weight change was defined as the difference between the present weight and the weight a year preceding breast cancer diagnosis. Percent of weight change was calculated as = [present weight (kg) - weight a year preceding breast cancer diagnosis (kg)]/weight a year preceding breast cancer diagnosis (kg) x100%. The changes in weight for the women were then categorized into >10% weight loss, 5-10% weight loss, no change (<5% weight loss and weight gain), 5-10% weight gain and >10% weight gain groups [26]. These weight change categories were chosen as they are commonly used for weight loss recommendation to reduce the risk of obesity and cancer. A reduction of 5-10% of body weight is likely to have a significant beneficial health effect on those who need to lose weight, even if the ideal weight was not achieved.

#### Other variables

Information were also collected on socio-demographic background (e.g. age, education level, employment status, marital status), reproductive and breast cancer history (e.g. time since diagnosis, menopausal status, type of treatments), family history of breast and other specific cancers.

### Statistical analysis

All data were analyzed using the *Statistical Package for the Social Sciences for Windows *(SPSS version 15) (Chicago, IL, USA). One way analysis of variance (ANOVA) and Chi-square analysis were used to examine weight change according to selected socio-demographic characteristics. The general linear model of analysis of covariance (ANCOVA) was used to test the differences in dietary intake, and physical activity by weight change groups with household income, years of education, age at diagnosis and time since diagnosis as covariates. The LSD (Least Significant Difference) test was applied to evaluate the statistically significant mean difference among the weight change groups.

## Results

Table 1 shows the socio-demographic characteristics, reproductive and breast cancer history of respondents. The median age of the women was 53 years. Among the women, more than half (57.1%) were Malay, 33.2% Chinese and 9.8% Indian. More than half had completed secondary education (51.9%) and 69.3% were currently unemployed. Over 80% of the women were post-menopausal at study entry. The mean duration of time from diagnosis to study entry was 4.86 years (95% CI: 4.50-5.21). About 41.0% of the women were diagnosed with cancer stage II, 31.8% stage I and 15.7% stage III. About 69% of the women reported a family history of cancer. The type of treatments that the women received varied depending on their stage of cancer at the time of diagnosis. Most women had undergone a mastectomy (79.6%) and chemotherapy (82.9%).

**Table 1:** Socio-demographic characteristics, reproductive and breast cancer history of women

Characteristics	Total n (%)	Mean (95% CI)
Age (years) *(Median)*		53.00
		
Ethnicity		
Malay	210 (57.1)	
Chinese	122 (33.2)	
Indian and Punjabi	36 (9.8)	
		
Marital status		
Single	18 (4.9)	
Married	304 (82.6)	
Divorced/Widowed	46 (12.5)	
		
Education years (years)		8.96 (8.56-9.36)
		
Employment status		
Employed	113 (30.7)	
Unemployed	255 (69.3)	
		
Monthly personal income (RM)		636.84 (500.32-768.12)
		
Monthly household income (RM)		2022.58 (1897.89-2426.08)
		
Menopausal status at study entry		
Pre-menopausal	70 (19.0)	
Post-menopausal	298 (81.0)	
		
Age at diagnosis (years)		48.53 (47.60-49.46)
		
Time since diagnosis (years)		4.86 (4.50-5.21)
		
Stage of cancer		
Stage I	117 (31.8)	
Stage II	151 (41.0)	
Stage III	58 (15.8)	
Not known	42 (11.4)	
		
Treatment		
Lumpectomy	62 (16.8)	
Mastectomy	293 (79.6)	
Radiotherapy	293 (79.6)	
Chemotherapy	305 (82.9)	
Hormonal therapy	159 (43.2)	
		
Family history of cancer		
Yes	115 (31.2)	
No	253 (68.8)	

Table 2 describes the weight status of respondents. The mean weight a year preceding breast cancer diagnosis, at diagnosis and current weight were 59.36 kg (95% CI: 58.17-60.55), 58.36 kg (95% CI: 57.51-59.95) and 62.09 kg (95% CI: 60.75-63.43), respectively. Nearly half of the women (49.2%) had normal BMI a year preceding breast cancer diagnosis. However, the percentage of women with normal BMI decreased to about 41% at study entry. Nearly two-thirds (65.7%) of the women were abdominally obese based on waist circumference. The mean weight change and percentage of weight change in women from a year preceding breast cancer diagnosis to study entry were 2.73 kg (95% CI: 1.90-3.55) and 5.17% (95% CI: 3.72-6.61), respectively. About 47.8% of women gained at least 5% of weight and about one-third (29.9%) had gained >10% of weight.

**Table 2:** Weight status of women with breast cancer

Weight information	Total	Mean (95% CI)
Weight a year preceding breast cancer diagnosis (kg)		59.36 (58.17-60.55)
		
Weight at time of diagnosis (kg)		58.36 (57.51-59.95)
		
Weight at study entry (kg)		62.09 (60.75-63.43)
		
Height (m)		1.55 (1.54-1.55)
		
Waist circumference at study entry (cm)^b^		84.81 (83.62-85.99)
Increased risk (≥80)	94 (25.5)	
Substantial risk (≥88)	148 (40.2)	
		
BMI a year preceding breast cancer diagnosis (kg/m^2^)^a^		24.82 (24.35-25.29)
Underweight (<18.5)	21 (5.7)	
Normal (18.5-24.9)	181 (49.2)	
Overweight (25.0-29.9)	122 (33.2)	
Obese (≥30.0)	44 (12.0)	
		
BMI at study entry (kg/m^2^)^a^		25.93 (25.41-26.45)
Underweight (<18.5)	20 (5.4)	
Normal (18.5-24.9)	151 (41.0)	
Overweight (25.0-29.9)	38 (37.5)	
Obese (≥30.0)	59 (16.0)	
		
Weight change from a year preceding diagnosis to study entry (kg)		2.73 (1.90-3.55)
>10 kg weight loss	15 (4.1)	
5-10 kg weight loss	39 (10.6)	
0-<5 kg weight loss	83 (22.6)	
0-<5 kg weight gain	105 (28.5)	
5-10 kg weight gain	71 (19.3)	
>10 kg weight gain	55 (14.9)	
		
Percentage of weight change from a year preceding diagnosis to study entry (%)		5.17 (3.72-6.61)
> 10% weight loss	40 (10.9)	
5-10% weight loss	41 (11.1)	
0-<5% weight loss	54 (14.7)	
0-<5% weight gain	57 (15.5)	
5-10% weight gain	66 (17.9)	
> 10% weight gain	110 (29.9)	

^a ^World Health Organization, (1998)

^b ^Increased risk of metabolic complications associated with obesity

^c ^Substantial risk of metabolic complications associated with obesity

Dietary intake and physical activity of women after breast cancer diagnosis are described in Table 3. The mean total energy intake of women was 1,350 kcal/day (95% CI: 1307.56-1391.84). The mean percentage of energy from carbohydrate, protein and fat were 56.02% (95% CI: 54.98-57.08), 16.38% (95% CI: 15.92-16.86) and 27.47% (95% CI: 26.61-28.34), respectively. Women reported an average of 6.09 daily servings of grains and cereals, 2.18 daily servings of meats and legumes, 0.76 daily servings of fruits, 1.18 daily servings of vegetables and 0.33 daily servings of dairy products, respectively. Most of the women (92.4%) did not currently consume alcohol. About 42% of the respondents had a high level of activity, 38.3% moderate and 19.6% low activity levels. Most respondents (86.1%) were engaged in work/domestic, transport related (61.4%) and recreational (41.0%) activities.

**Table 3:** Dietary intake and physical activity of women after breast cancer diagnosis

	Total	Mean (95% CI)	
Dietary intake			
Total energy intake (kcal/day)		1350 (1307.56-1391.84)	
Percentage of energy from carbohydrate (%)		56.02 (54.98-57.08)	
Percentage of energy from protein (%)		16.38 (15.92-16.86)	
Percentage of energy from fat (%)		27.47 (26.61-28.34)	
Number of servings from grains and cereals ^a^		6.09 (5.91-6.35)	
Number of servings from meats ^b^		2.18 (2.04-2.29)	
Number of servings from fruits		0.76 (0.61-0.82)	
Number of serving from vegetables		1.18 (1.08-1.24)	
Number of serving from dairy products ^c^		0.33 (0.27-0.39)	
Alcohol intake			
Yes	28 (7.6)		
No	340 (92.4)		
			
Physical activity			
Total physical activity (MET-min/week)		3529.56	
Low	72 (19.6)	(3052.64-3986.36)	
Moderate	141 (38.3)		
High	155 (42.1)		
			
Activities at work/domestic (min/day)		79.15 (68.53-89.76)	
Did work activity	317 (86.1)		
No work activity	51 (13.9)		
			
Travel to and from places (min/day)		15.97 (12.68-17.69)	
Did travel activity	226 (61.4)		
No travel activity	142 (38.6)		
			
Recreational activities (min/day)		18.21 (14.85-21.58)	
Did recreational activity	151 (41.0)		
No recreational activity	169 (45.9)		
			
Sedentary behavior ^d ^(min/day)		206.62 (191.92-221.32)	

^a ^grains and cereals-grain/cereal/tubers based on Malaysian Dietary Guidelines (2010)

^b ^meats-meat/fish/poultry/legumes based on Malaysian Dietary Guidelines (2010)

^c ^dairy products-milk/dairy products based on Malaysian Dietary Guidelines (2010)

^d ^Sitting or reclining at work, at home, getting to and from places including time spent on sitting at desk, travelling in car, bus or watching television but do not include time spent sleeping

Among socio-demographic and cancer characteristics of women, only BMI a year before diagnosis was significantly different by weight change groups (Table 4). Women with weight gains have significantly lower pre-diagnosis BMI than those with weight loss or stable weight (Table 4). Dietary intake and physical activity of women by weight changes are presented in Table 5. The number of servings for fruits and vegetables and dairy products differed significantly among the weight change groups. The least number of servings for fruits and vegetables was reported for the >10% weight gain group (1.58 servings/day; 95% CI: 1.36-1.82) compared to other weight change groups. In contrast, the >10% weight gain group was found to have the greatest number of servings for dairy products compared to the >10% weight loss group (p < 0.05), although none of the groups achieved the recommended 2-3 servings/day of milk and dairy products. Women in all weight change groups did not differ significantly in physical activity.

**Table 4:** Socio-demographic and cancer characteristics of women by weight change groups

Characteristics	Weight changes groups since diagnosis			Mean (95% CI)		p-value
	>10%	5-10%	No change*	5-10%	>10%	
	weight loss	weight loss		weight gain	weight gain	
	(n = 40)	(n = 41)	(n = 114)	(n = 60)	(n = 113)	
Age at diagnosis (years)	50.05 (47.45-52.65)	51.02 (48.17-53.87)	49.19 (47.34-51.05)	47.28 (45.11-49.46)	47.05 (45.47-48.63)	0.06
						
Ethnicity						
Malay	24 (58.5)	20 (48.8)	60 (52.6)	34 (56.7)	72 (19.6)	0.34
Non-Malay	17 (41.5)	21 (51.2)	54 (47.4)	26 (43.3)	40 (35.7)	
						
Marital status						0.43
Married	35 (85.4)	36 (87.8)	90 (78.9)	53 (88.3)	90 (80.4)	
Others**	6 (14.6)	5 (12.2)	24 (21.1)	7 (11.7)	22 (19.6)	
						
Education (years)	8.56 (7.26-9.86)	8.54 (7.30-9.78)	8.68 (7.93-9.44)	9.92 (8.93-10.90)	9.03 (8.34-9.71)	0.28
Secondary and lower	22 (53.7)	24 (58.5)	58 (50.9)	22 (36.7)	58 (51.8)	
Tertiary and others	19 (46.3)	17 (41.5)	56 (49.1)	38 (63.3)	54 (48.2)	
						
Employment status						0.86
Employed	10 (24.4)	12 (29.3)	34 (29.8)	20 (33.3)	37 (33.0)	
Unemployed	31 (75.6)	29 (70.7)	80 (70.2)	40 (66.7)	75 (67.0)	
						
Monthly household income (RM)	1756.10 (1246.58-2265.61)	2002.63 (1252.71-2752.56)	1967.76 (1563.28-2372.25)	2968.67 (2089.90-3847.44)	2134.45 (1620.90-2647.99)	0.10
						
Menopausal status at study entry						0.10
Pre-menopausal	3 (7.3)	4 (9.8)	24 (21.1)	14 (23.3)	25 (22.3)	
Post-menopausal	38 (92.7)	37 (90.2)	90 (78.9)	46 (76.7)	87 (77.7)	
Time since diagnosis (years)	4.63 (3.41-5.86)	5.20 (3.74-6.65)	4.67 (4.03-5.31)	4.48 (3.69-5.28)	5.21 (4.65-5.78)	0.59
						
Stage of cancer						0.89
Stage I	9 (24.3)	12 (31.6)	36 (37.5)	22 (40.7)	38 (37.6)	
Stage II	21 (56.8)	19 (50.0)	43 (44.8)	22 (40.7)	46 (45.5)	
Stage III	7 (18.9)	7 (18.4)	17 (17.7)	10 (18.5)	17 (16.8)	
						
BMI a year preceding diagnosis*** (kg/m^2^)	26.62 (24.86-28.37)	25.96 (24.45-27.47)	25.18 (24.33-26.04)	24.50 (23.38-25.62)	23.54 (22.78-24.29)	0.001****^a, b, c, d^

*0-5% weight loss or weight gain

**Single, widowed or divorced

***Body mass index a year preceding breast cancer diagnosis

****p < 0.05

^a ^A significant difference between >10% weight loss and 5-10% weight gain groups, at p < 0.05.

^b ^A significant difference between >10% weight loss and >10% weight gain groups, at p < 0.05.

^c ^A significant difference between 5-10% weight loss and >10% weight gain groups, at p < 0.05.

^d ^A significant difference between no change and >10% weight gain groups, at p < 0.05.

**Table 5:** Lifestyle behaviors of women by weight change groups

		Weight change since diagnosis	Mean (95% CI)			p-value
	>10%	5-10%	No change*	5-10%	>10%	
	weight loss	weight loss		weight gain	weight gain	
	(n = 40)	(n = 41)	(n = 114)	(n = 60)	(n = 113)	
Total energy intake (kcal/day)	1344	1408	1330	1377	1335	0.82
Percentage of energy intake from carbohydrate (%)	55.76 (52.66-59.03)	55.20 (52.00-58.39)	56.79 (54.88-58.70)	55.73 (53.08-58.39)	55.81 (53.85-57.72)	0.90
Percentage of energy intake from protein (%)	16.15 (14.62-17.45)	17.41 (15.99-18.83)	15.76 (14.91-16.61)	16.66 (15.48-17.84)	16.59 (15.77-17.49)	0.30
Percentage of energy intake from fat (%)	28.10 (25.52-30.75)	27.35 (24.73-29.97)	27.50 (25.93-29.07)	27.49 (25.31-29.67)	27.27 (25.66-28.83)	0.98
Number of servings from grains and cereals	5.99 (5.40-6.70)	6.88 (6.23-7.54)	6.23 (5.84-6.62)	5.70 (5.15-6.24)	6.04 (5.62-6.41)	0.87
Number of servings from meats	2.08 (1.72-2.45)	2.49 (2.13-2.85)	2.12 (1.91-2.34)	2.13 (1.83-2.44)	2.16 (1.94-2.38)	0.49
Number of servings from fruits and vegetables	2.06 (1.66-2.41)	2.12 (1.74-2.50)	2.00 (1.77-2.22)	1.94 (1.62-2.26)	1.58 (1.36-1.82)	0.04**^a, b, c^
Number of serving from dairy products	0.19 (0.01-0.36)	0.31 (0.13-0.49)	0.33 (0.22-0.44)	0.31 (0.17-0.46)	0.41 (0.30-0.52)	0.03**^a^
						
Alcohol intake						0.12
Yes	2 (7.1)	1 (3.6)	12 (42.9)	6 (21.4)	7 (25.0)	
No	38 (11.2)	40 (11.8)	102 (30.0)	54 (15.9)	106 (31.2)	
						
Total physical activities (MET-min/week)	4436.71 (3112.63, 5602.65)	4246.85 (2996.98, 5494.10)	2817.77 (2071.49, 3563.63)	3753.38 (2717.08, 4790.58)	3351.85 (2616.35, 4126.77)	0.15
Low	5 (12.5)	9 (22.0)	28 (24.6)	12 (20.0)	18 (15.9)	0.16
Moderate	16 (40.0)	11 (26.8)	47 (41.2)	17 (28.3)	50 (44.2)	
High	19 (47.5)	21 (51.2)	39 (34.2)	31 (51.7)	45 (39.8)	

Adjusted for household income, years of education, age at diagnosis and time since diagnosis

* 0-5% weight loss or weight gain

** p < 0.05

^a ^A significant difference between >10% weight loss and >10% weight gain groups, at p < 0.05.

^b ^A significant difference between 5-10% weight loss and >10% weight gain groups, at p < 0.05.

^c ^A significant difference between no change and >10% weight gain groups, at p < 0.05.

## Discussion

Weight gain is a common observation among women with breast cancer. This study supports the existing evidence that women tend to gain weight after breast cancer diagnosis. A significant gain in weight amounting to 2-3 kg after diagnosis of breast cancer was found in this study. Although some aspects of weight gain pattern among the breast cancer survivors in our study were similar to those observed in other studies [12,27,28], the magnitude of weight gain differed. The magnitude of weight gain observed from pre-diagnosis to study entry was somewhat similar (2.4 kg) to that reported by Caan et al., (2006) [17], but was lower (3.17 kg) than that reported in the WHEL study [29]. In both studies, the mean duration between time of diagnosis and study entry was similar (4-5 years) to our study.

Overweight and obesity were already prevalent in over 40% of the women prior to diagnosis, however more women were overweight and obese at study entry. Similar to Rock et al. (1999) [7], we also showed that women with higher weight gains had significantly lower pre-diagnosis BMI than those with lower weight gains and stable weight. A higher body mass index (BMI) in women after breast cancer diagnosis will increase the risk for recurrence and lower the survival [7,12]. Kroenke et al. (2005) [30] showed that breast cancer survivors who increased their body mass index by 0.5-2.0 kg/m^2 ^had a risk ratio of recurrence of 1.4 and those who gained more than 2.0 kg/m^2 ^had a risk ratio of 1.53, with both groups having significantly higher all-cause mortality compared to survivors with a stable weight. The clinical significance of weight gain (2.7 kg) observed in the present study is not known, however the calculated increase (1 year before diagnosis to study entry) in body mass index is 1.11 kg/m^2 ^which if based on previous studies could have significant effect on cancer recurrence and survival. About two-thirds of the women (65.8%) in this present study had at risk waist circumference. Having a higher BMI and waist circumference could put these women at risk of cardiovascular diseases, diabetes and cancer recurrence [31].

We found that women who gained weight after breast cancer diagnosis had lower fruit and vegetable intakes compared to those who lost weight. Although the weight loss groups had a higher intake of fruit and vegetables, the number of servings still did not meet the recommendation of 3-5 servings daily. Increasing the consumption of fruits and vegetables may reduce the risk of overweight/obesity as well as facilitate the slowing down of weight gain, especially among overweight and obese adults [32]. However, the Women's Healthy Eating and Living (WHEL) study, a randomized controlled dietary intervention study that examined the effect of plant-based diets without a specific energy restriction among breast cancer survivors, showed that diets high in fruits, vegetables and fiber were not significantly associated with weight change [33]. Although the mechanism underlying fruit and vegetable intake and body weight is still uncertain, it is known that fruits and vegetables are generally low in energy density, which could influence energy intake [34]. Consumption of ≥9 servings of fruit and vegetables is associated with lower energy density as well as a lower prevalence of obesity even for diets high in fat [35]. Furthermore, fruits and vegetables have a high water and fiber content, which, in turn, promotes satiety. Therefore, women who consume more fruits and vegetables are more likely to lose weight compared to those who consume less fruit and vegetables.

Adequate consumption of dairy products is encouraged because they are rich sources of essential nutrients that are important for bone health. The present study found that women who gained > 10% of weight consumed significantly more servings of dairy products than those who lost > 10% of weight after breast cancer diagnosis; although none of the groups met the recommended intake of 1-3 servings of dairy products. A plausible explanation for the low dairy product intake among these women is that many believed milk and dairy products are high in saturated fat and cholesterol, and contain carcinogens that can cause cancer recurrence. Although we did not examine changes in dietary intake before and after cancer diagnosis, it is possible that these women could have stopped or reduced the intake of dairy products since cancer diagnosis. Skeie et al. (2009) [36] also reported that breast cancer survivors reduced their milk consumption after breast cancer diagnosis. However, the Women's Intervention Nutrition Study (WINS) showed that breast cancer survivors had 1.6-1.8 servings per day of dairy products [37]. Several studies [38,39] have reported that low fat dairy products could prevent weight gain and promote weight loss among healthy adult population. An adequate dairy consumption in conjunction with controlled/reduced caloric intake may be associated with weight regulation [39-41] and reduced adiposity [42,43], indicating that dairy intake may be valuable for weight maintenance.

Most of the women (80.4%) in the present study had moderate and high physical activity level. This physical activity pattern is consistent with the findings of a study among United States women that showed 70% of women with breast cancer had moderate-intensity physical activity while 10% had high-intensity physical activity [19]. However, another study [44] among breast cancer survivors found that the majority (74.6%) had low intensity physical activity and only 25.4% had moderate and high intensity physical activity. In addition, cancer survivors have also been reported to be more active than non-cancer controls [45]. Perhaps, they are more likely to adopt healthier lifestyle changes [46], which could explain why most of the women in the present study had at least a moderate-intensity physical activity.

This study has several limitations. The small sample size of this study may not be representative of the population of women with breast cancer in Malaysia. Despite the recruitment of respondents from hospitals and breast cancer support groups throughout the country, there could still be differences in socio-demographic, reproductive and other characteristics. Another limitation is that study participation was voluntary which could result in selection bias in that those who are weight or health conscious would participate in the study. This study used self-reported weight a year preceding breast cancer diagnosis, which is subject to recall error and consequently could affect the calculation and classification of weight change. However, self-reported weight has been shown to be accurate [47] and most epidemiological studies have used self-reported weight and weight change data [48,49]. Self-reported measures also tend to overestimate desirable health behaviors such as physical activity and intake of fruits and vegetables. Being overweight and diagnosed with breast cancer may prompt women to over-report healthy behaviors. In contrast, undesirable health behaviors (i.e. smoking, sedentary lifestyle, high dietary fat intake) will be under-reported. This study was cross-sectional in that the information on factors associated with weight change could not imply cause and effect relationship. A longitudinal study design is most appropriate to identify changes in lifestyle behaviors that cause weight loss or gain in cancer survivors. As we did not measure weight, dietary intake and physical activity level at other times (e.g. every year after breast cancer diagnosis), the selection of period to measure these variables could contribute to different results. For example, it is possible that although the study only measured weight status at fixed periods, i.e. a year before breast cancer diagnosis, at diagnosis and at study entry, women will initially lose or gain weight at different times. It is also possible that women make lifestyle changes from time to time and that their current diets or physical activity may be better or healthier compared to their earlier practices. Despite these limitations, this is the first study to investigate weight change before and after diagnosis in a sample of Malaysian women diagnosed with breast cancer.

## Conclusion

The high proportion of breast cancer survivors experiencing weight gain in this study suggests that efforts are needed to encourage breast cancer survivors to achieve or maintain healthy body weight. Understanding lifestyle behaviors i.e. dietary intake and physical activity of women diagnosed with breast cancer and with varying degrees of weight change is essential for the development and targeting of effective strategies to improve health and survival of breast cancer survivors. As the intake of fruits, vegetables and dairy products of women in this study are still below the recommended servings per day, dietary strategies should target all breast cancer survivors, regardless of their weight status. Despite limited evidence on how lifestyle behaviors prevents cancer recurrence or prolongs life, a diagnosis of breast cancer should be a starting point for women to have healthful diets and regular physical activity.

## Competing interests

The authors declare that they have no competing interests.

## Authors' contributions

YHY designed the study, conducted data collection, performed and interpreted the analyses and prepared the manuscript; ZMS designed the study, supervised and assisted data collection, analysed and interpreted of study findings, assisted in the preparation of the manuscript and approved the final manuscript; CYM, ZH, MK, RMY, ZO, NS and YHW supervised and assisted in study design and data collection. All authors read and revised the manuscript.

## Pre-publication history

The pre-publication history for this paper can be accessed here:

http://www.biomedcentral.com/1471-2458/11/309/prepub

## References

[B1] World Health Organization World Health Statistics 2008 2008 Geneva: World Health Organization

[B2] Lim GCC Halimah Y Lim TO First Report of the National Cancer Registry: Cancer Incidence in Malaysia 2002 2003 Kuala Lumpur: National Cancer Registry

[B3] Zainal AO Zainudin MA Nor Saleha IT Malaysian Cancer Statistics-Data and Figure Peninsular Malaysia 2006 2006 National Cancer Registry. Ministry of Health Malaysia: Kuala Lumpur

[B4] Sant M Aareleid T Berrino F Lasota MB Carli PM Faivre J Grosclaude P Hedelin G Matsuda T Mollers H Moller T Verdecchia A Capocaccia R Gatta G Micheli A Santaquilani M Roazzi P Lisi D EUROCARE Working Group EUROCARE-3: survival of cancer patients diagnosed 1990-94--results and commentary Annals of Oncology 2003 14 5 v61 v118 10.1093/annonc/mdg754 14684501

[B5] American Cancer Society (ACS) Breast Cancer Facts & Figures 2007-2008 2007 American Cancer Society, Atlanta, Georgia21347935

[B6] Yip CH Nur Aishah MT Ibraham M Epidemiology of breast cancer in Malaysia Asian Pacific Journal of Cancer Prevention 2006 7 369 374 17059323

[B7] Rock CL Flatt SW Newman V Caan BJ Haan MN Stefanick ML Faerber S Pierce JP Factors associated with weight gain in women after diagnosis of breast cancer Journal of the American Dietetic Association 1999 99 10 1212 1221 10.1016/S0002-8223(99)00298-9 10524383

[B8] Halbert CH Weathers B Esteve R Audrain-McGovern J Kumanyika S DeMichele A Barg F Experiences with weight change in African-American breast cancer survivors The Breast Journal 2008 14 2 182 187 10.1111/j.1524-4741.2007.00551.x 18282235

[B9] Heideman WH Russell NS Gundy C Rookus MA Voskuil DW The frequency, magnitude and timing of post-diagnosis body weight gain in Dutch breast cancer survivors European Journal of Cancer 2009 45 1 119 126 10.1016/j.ejca.2008.09.003 18930387

[B10] Makari-Judson G Judson CH Mertens WC Longitudinal patterns of weight gain after breast cancer diagnosis: Observations beyond the first year The Breast Journal 2007 13 3 258 265 10.1111/j.1524-4741.2007.00419.x 17461900

[B11] Rock CL Demark-Wahnefried W Nutrition and survival after the diagnosis of breast cancer: a review of the evidence Journal of Clinical Oncology 2002 20 3302 3306 10.1200/JCO.2002.03.008 12149305 PMC1557657

[B12] Irwin ML McTiernan A Baumgartner RN Baumgartner KB Bernstein L Gilliland FD Ballard-Barbash R Changes in body fat and weight after a breast cancer diagnosis: Influence of demographic, prognostic, and lifestyle factors Journal of Clinical Oncology 2005 23 4 774 782 10.1200/JCO.2005.04.036 15681521 PMC3000612

[B13] Salminen EK Lagstrom HK Heikkila SP Salminen SJ Does breast cancer patients' change dietary habits? European Journal of Clinical Nutrition 2000 54 844 848 10.1038/sj.ejcn.1601103 11114679

[B14] Maunsell E Drolet M Brisson J Robert J Deschenes L Dietary chang after breast cancer: extent, predictors, and relation with psychological distress American Society of Clinical Oncology 2002 20 1017 1025 10.1200/JCO.2002.20.4.1017 11844825

[B15] Thomson CA Flatt SW Rock CL Ritenbaugh C Newman VA Pierce JP Increased fruit, vegetable and fiber intake and lower fat intake reported among women previously treated for invasive breast cancer Journal of the American Dietetic Association 2002 102 801 808 10.1016/S0002-8223(02)90180-X 12067045

[B16] Wayne SJ Lopez ST Butler LM Baumgartner KB Baumgartner RN Ballard-Barbash R Changes in Dietary Intake after Diagnosis of Breast Cancer Journal of the American Dietetic Association 2004 104 1561 1568 10.1016/j.jada.2004.07.028 15389414

[B17] Irwin ML McTiernan A Bernstein L Gilliland FD Baumgartner R Baumgartner K Ballard-Barbash R Physical activity levels among breast cancer survivors Medical & Science in Sports & Exercise 2004 36 1484 1491 15354027 PMC3000611

[B18] Harrison S Hayes SC Newman B Level of physical activity and characteristics associated with change following breast cancer diagnosis and treatment Psycho-Oncology 2009 18 4 387 394 10.1002/pon.1504 19117320

[B19] Emaus A Veierod MB Tretli S Finstad SE Selmer R Furberg AS Bernstein L Thune I Metabolic profile, physical activity, and mortality in reast cancer patients Breast Cancer Research Treatment 2010 121 3 651 660 10.1007/s10549-009-0603-y 19882245

[B20] First Data Bank 2005 Nutritionist Pro. San Bruno, CA 94066

[B21] Tee ES Ismail MN Nasir MA Khatijah I Nutrient Composition of Malaysia Foods 1997 Kuala Lumpur: Institute of Medical Research

[B22] Department of Nutrition, Ministry of Health Singapore food facts Singapore 1999

[B23] Puwastien P Burlingame B Raroengwichit Sungpuag P ASEAN Food Composition Tables 2000 2000 Institute of Nutrition: Mahidol University, Thailand

[B24] Global Physical Activity Questionnaire (GPAQ) http://www.who.int/chp/steps/GPAQ/en/

[B25] World Health Organization (WHO) Physical status: the use and interpretation of anthropometry 1995 Report of a WHO Expert Committee. WHO Technical Report Series 854. Geneva 8594834

[B26] Doyle C Kushi LH Byers T Nutrition and physical activity during and after cancer treatment: an American Cancer Society guide for informed choices Cancer Journal for Clinicians 2006 56 6 323 353 10.3322/canjclin.56.6.323 17135691

[B27] Camoriano J Loprinzi C Ingle J Therneau T Krook J Veeder M Weight change in women treated with adjuvant therapy or observed following mastectomy for node-positive breast cancer Journal of Clinical Oncology 1990 8 1327 1334 2199619 10.1200/JCO.1990.8.8.1327

[B28] Gu K Chen XI Zheng Y Chen Z Zheng W Lu W Shu XO Weight change patterns among breast cancer survivors: results from the Shanghai breast cancer survival study Cancer Causes and Control 2009 21 4 621 629 20043201 10.1007/s10552-009-9491-z PMC3770524

[B29] Pierce JP Faerber S Wright FA Rock CL Newman V Flatt SW Kealey S Jones VE Caan BJ Gold EB Haan M Hollenbach KA Jones L Marshall JR Ritenbaugh C Stefanick ML Thomson C Wasserman L Natarajan L Thomas RG Gilpin EA the Women's Healthy Eating and Living (WHEL) Study Group A randomized trial of the effect of a plant-based dietary pattern on additional breast cancer events and survival: the Women's Healthy Eating and Living (WHEL) Study Controlled Clinical Trials 2002 23 6 728 756 10.1016/S0197-2456(02)00241-6 12505249

[B30] Kroenke CH Chen WY Rosner B Holmes MD Weight, weight gain, and survival after breast cancer diagnosis Journal of Clinical Oncology 2005 23 1370 1378 10.1200/JCO.2005.01.079 15684320

[B31] Caan BJ Kwan ML Hartzell G Castillo A Slattery ML Sternfeld B Weltzien E Pre-diagnosis body mass index, post-diagnosis weight and prognosis among women with early stage breast cancer Cancer Causes Control 2008 19 1319 1328 10.1007/s10552-008-9203-0 18752034 PMC2925505

[B32] Sartorelli DS Franco LJ Cardoso MA High intake of fruits and vegetables predicts weight loss in Brazilian overweight adults Nutrition Research 2008 28 233 238 10.1016/j.nutres.2008.02.004 19083413

[B33] Demark-Wahnefried W Hars V Conaway MR Havlin K Rimer BK McElveen G Winer EP Reduced rates of metabolism and decreased physical activity in breast cancer patients receiving adjuvant chemotherapy American Journal of Clinical Nutrition 1997 65 1495 1501 9129482 10.1093/ajcn/65.5.1495

[B34] Bell EA Castellanos VH Pelkman CL Thorwart ML Rolls BJ Energy density of foods affects energy intake in normal-weight women American Journal of Clinical Nutrition 1998 67 412 420 9497184 10.1093/ajcn/67.3.412

[B35] Ledikwe JH Blanck HM Khan LK Serdula MK Seymour JD Tohill DC Rolls BJ Dietary energy density is associated with energy intake and weight status in US adults American Journal of Clinical Nutrition 2006 83 6 1362 1368 16762948 10.1093/ajcn/83.6.1362

[B36] Skeie G Hjartaker A Braaten T Lund E Dietary change among breast and colorectal cancer survivors and cancer-free women in the Norwegian women and cancer cohort study Cancer Causes Control 2009 20 1955 1966 10.1007/s10552-009-9390-3 19565341

[B37] Chlebowski RT Blackburn GL Thomson CA Nixon DW Shapiro A Hoy MK Goodman MT Giuliano AE Karanja N McAndrew P Butler CHJ Merkel D Kristal A Caan B Michaelson R Vinciguerra V Prete S Winkler M Hall R Simon M Winters BL Elashoff RM Dietary fat reduction and breast cancer outcome: Interim Efficacy results from the women's intervention nutrition study Journal of the National Cancer Institute 2006 98 24 1769 1776 10.1093/jnci/djj494 17179478

[B38] Lau EMC Woo J Lam V Hong A Milk supplementation of the diet by ostmenopausal Chinese women on a low calcium intake retards bone loss Journal of Bone and Mineral Research 2001 16 9 1704 1709 10.1359/jbmr.2001.16.9.1704 11547841

[B39] Brooks BM Rajeshwari R Nicklas TA Yang SJ Berenson GS Association of calcium intake, dairy product consumption with overweight status in young adults (1995-1996): The Bogalusa Heart Study Journal of the American College of Nutrition 2006 25 6 523 532 17229900 10.1080/07315724.2006.10719568 PMC2769990

[B40] Pereira MA Jacobs DR Van Horn L Slattery ML Kartashov AI Ludwig DS Dairy consumption, obesity, and the insulin resistance syndrome in young adults: The CARDIA Study The Journal of the American Medical Association 2002 287 2081 2089 10.1001/jama.287.16.2081 11966382

[B41] Mirmiran P Esmaillzadeh A Azizi F Dairy consumption and body mass index: An inverse relationship International Journal of Obesity (Lond) 2005 29 115 121 10.1038/sj.ijo.0802838 15534616

[B42] Zemel MB Richards J Mathis S Milstead A Gebhardt L Silva E Dairy augmentation of total and central fat loss in obese subjects International Journal of Obesity and Related Metabolic Disorders 2005 29 391 397 10.1038/sj.ijo.0802880 15672113

[B43] Zemel MB Richards J Milstead A Campbell P Effects of calcium and dairy on body composition and weight loss in African-American adults Obesity Research 2005 13 1218 1225 10.1038/oby.2005.144 16076991

[B44] Abdul-Samad AA Al-Kamil EA Al-Sodani AH Breast Cancer and Selected Lifestyle Variables: A Case-Control Study Bahrain Medical Bulletin 2009 31 4 1 10

[B45] Bellizi KM Rowland JH Jeffery DD McNeel T Health Behaviors of Cancers Survivors: Examining Opportunities for Cancer Control Intervention Journal of Clinical Oncology 2005 23 8884 8893 10.1200/JCO.2005.02.2343 16314649

[B46] Parkin DM Bray F Ferlay J Pisani P Global cancer statistics, 2002 Cancer Journal for Clinicians 2005 55 74 108 10.3322/canjclin.55.2.74 15761078

[B47] Spencer EA Appleby PN Davey GK Key TJ Validity of self-reported height and weight in 4808 EPIC-Oxford participants Public Health Nutrition 2002 5 561 565 10.1079/PHN2001322 12186665

[B48] Loi S Milne RL Friedlander ML McCredie MRE Giles GG Hopper JL Phillips KA Obesity and outcomes in premenopausal and postmenopausal breast cancer Cancer Epidemiology, Biomarkers & Prevention 2005 14 7 1686 1691 10.1158/1055-9965.EPI-05-0042 16030102

[B49] Whiteman MK Hillis SD Curtis KM McDonal JA Wingo PA Marchbanks PA Body mass and mortality after breast cancer diagnosis Cancer Epidemiology, Biomarkers & Prevention 2009 14 8 2009 2014 16103453 10.1158/1055-9965.EPI-05-0106

